# Uncovering the genetic basis for enhanced mushroom flavor in *Quercus fabri* through genome sequencing and metabolic profiling

**DOI:** 10.1093/hr/uhaf156

**Published:** 2025-07-09

**Authors:** Liwen Wu, Yuqing Cai, Chenggang Jiang, Xiang Shi, Shifa Xiong, Yicun Chen, Yangdong Wang

**Affiliations:** State Key Laboratory of Tree Genetics and Breeding, Chinese Academy of Forestry, No. 1 Dongxiaofu, Xiangshan Road, Beijing Haidian District, Beijing 100091, China; Research Institute of Subtropical Forestry, Chinese Academy of Forestry, No. 73 Daqiao Road, Hangzhou Fuyang District, Zhejiang 311400, China; State Key Laboratory of Tree Genetics and Breeding, Chinese Academy of Forestry, No. 1 Dongxiaofu, Xiangshan Road, Beijing Haidian District, Beijing 100091, China; Research Institute of Subtropical Forestry, Chinese Academy of Forestry, No. 73 Daqiao Road, Hangzhou Fuyang District, Zhejiang 311400, China; State Key Laboratory of Tree Genetics and Breeding, Chinese Academy of Forestry, No. 1 Dongxiaofu, Xiangshan Road, Beijing Haidian District, Beijing 100091, China; Research Institute of Subtropical Forestry, Chinese Academy of Forestry, No. 73 Daqiao Road, Hangzhou Fuyang District, Zhejiang 311400, China; State Key Laboratory of Tree Genetics and Breeding, Chinese Academy of Forestry, No. 1 Dongxiaofu, Xiangshan Road, Beijing Haidian District, Beijing 100091, China; Research Institute of Subtropical Forestry, Chinese Academy of Forestry, No. 73 Daqiao Road, Hangzhou Fuyang District, Zhejiang 311400, China; State Key Laboratory of Tree Genetics and Breeding, Chinese Academy of Forestry, No. 1 Dongxiaofu, Xiangshan Road, Beijing Haidian District, Beijing 100091, China; Research Institute of Subtropical Forestry, Chinese Academy of Forestry, No. 73 Daqiao Road, Hangzhou Fuyang District, Zhejiang 311400, China; State Key Laboratory of Tree Genetics and Breeding, Chinese Academy of Forestry, No. 1 Dongxiaofu, Xiangshan Road, Beijing Haidian District, Beijing 100091, China; Research Institute of Subtropical Forestry, Chinese Academy of Forestry, No. 73 Daqiao Road, Hangzhou Fuyang District, Zhejiang 311400, China; State Key Laboratory of Tree Genetics and Breeding, Chinese Academy of Forestry, No. 1 Dongxiaofu, Xiangshan Road, Beijing Haidian District, Beijing 100091, China; Research Institute of Subtropical Forestry, Chinese Academy of Forestry, No. 73 Daqiao Road, Hangzhou Fuyang District, Zhejiang 311400, China

## Abstract

*Quercus fabri* is a common timber oak tree species widely distributed in subtropical areas of China. In this study, we presented a chromosome-scale reference genome assembly of *Q*. *fabri* achieved by integrating PacBio Sequel II, DNBseq™, and Hi-C sequencing platforms, and the results indicated the *Q*. *fabri* genome has a size of 836.74 Mb. Through the analysis of significantly expanded gene families, we identified that many of the top-ranked KEGG pathways are associated with amino acid metabolism. Subsequently, we performed an amino acid metabolic profile analysis on *Q*. *fabri* and related species, including *Quercus aliena*, *Quercus acutissima*, and *Quercus variabilis*. The findings revealed that the content of amino acids in *Q*. *fabri* was significantly higher than that in the other three oak species. Additionally, we found a significantly higher content of flavor amino acids, such as glutamic acid (Glu), aspartic acid (Asp), and glycine (Gly), in *Q*. *fabri*. Considering these results, we designed experiments to assess the nutrient content in mushrooms cultivated from the four oak trees. The results indicated that the total amino acid and protein content of mushrooms cultivated using *Q*. *fabri* as a substrate was significantly greater than that of mushrooms grown on the other three oak species. This characteristic may explain why *Q*. *fabri* wood is particularly effective as a substrate for cultivating more flavorful mushrooms. This study presents the complete genome and evolutionary information of *Q. fabri*, and integrates metabolic profiling to explore the underlying reasons for the enhanced flavor of mushrooms cultivated from it.

## Introduction

Among the angiosperms, tree species from the Fagaceae family hold significant importance and are prominent constituents of temperate, subtropical, and even tropical forest communities in the Northern Hemisphere [1]. Globally, there are eight genera within the Fagaceae family, with seven of these found in China: *Quercus*, *Lithocarpus*, *Castanopsis*, *Castanea*, *Fagus*, *Cyclobalanopsis*, and *Trigonobalanus* [2]. *Quercus*, which boasts the highest species count, is mainly distributed throughout Asia, Europe, Africa, and the Americas, serving as a crucial genus within forest ecosystems [3, 4]. Species within the *Quercus* genus predominantly contribute to forest formation, playing vital roles in the conservation of water and soil. The wood of *Quercus* species is durable and resistant to pressure, decay, and moisture, making it a preferred material in manufacturing vehicles, ships, construction, furniture, and various other applications [5, 6]. In natural forest ecosystems, *Quercus* could form stable symbiotic relationships with some specific edible fungi through ectomycorrhizal associations. It is considered evidence of a symbiotic mechanism that *Tuber melanosporum* colonizes the roots of the oak seedlings including *Quercus ilex*, *Quercus faginea*, and *Quercus acutissima*. Notably, this robust wood material serves as a premier cultivation substrate for edible fungi cultivation, with both *Quercus* log segments and wood chips supporting fungal growth through controlled nutrient release [7, 8]. In addition, the smaller branches of *Quercus* trees can also be crushed and packed into culture media to cultivate mushrooms, which enhances their flavor [9, 10].

**Figure 1:**
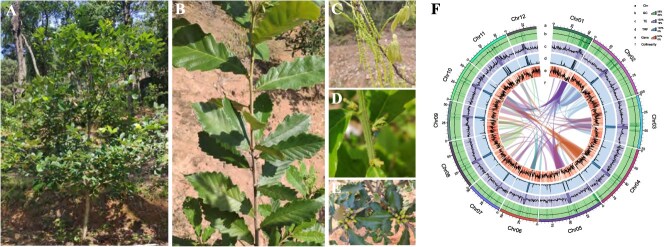
Characteristics of various parts of the *Q. fabri* and characteristics of the *Q. fabri* genome. **A**. The whole 5-year-old *Q. fabri* tree. **B**. *Q. fabri* branches and leaves. **C**. *Q. fabri* male flowers. **D**. *Q. fabri* female flowers. **E**. *Q. fabri* fruits. **F**. *Q. fabri* genome circos diagram (a represents the 12 chromosomes in *Q. fabri*, b represents guanine-cytosine (GC) density, c represents transposable element (TE) density, d represents tandem repeat (TRF) density, e represents gene density, and f represents collinearity).

Although the *Quercus* genus is readily identifiable, classification at the species level is often complicated by frequent natural hybridization and morphological similarity. These issues lead to overlapping traits and ambiguous taxonomic boundaries [11]. For instance, the oak species *Q*. *acutissima* and *Quercus variabilis* exhibit very similar morphological traits, primarily differentiated by the presence of white hairs on the underside of their leaves. However, their sympatric distribution promotes frequent hybridization, blurring the phenotypic boundaries between them [12]. Likewise, *Quercus liaotungensis* and *Quercus mongolica* share similar habitats and morphology, with the latter distinguished primarily by nodular protrusions on the small bracts of its cupule. While some consider *Q. liaotungensis* as a variant of *Q. mongolica*, molecular evidence based on isoenzyme and AFLP markers supports its classification as a distinct species. These enduring classification challenges highlight the growing reliance on genomic tools [13–15]. Though traditional morphology and genetic markers with plastid sequence data offer improved resolution, modern approaches leveraging phylogenomic data now enable detection of ancient hybridization patterns, precise measurement of introgression, and differentiation between convergent traits versus shared ancestry.

Molecular biological techniques frequently represent the most efficient approach for addressing significant biological issues related to *Quercus* species. In order to carry out molecular biological evaluations of various *Quercus* species, it is essential to possess an understanding of their genomic data. Currently, the complete genomic information for *Quercus robur*, *Quercus suber*, *Quercus lobata*, *Q. acutissima*, *Q. variabilis*, and *Q. mongolica* has been made available [6, 12, 16–19]. *Quercus robur* was the first oak to have its genome fully sequenced, while *Q. mongolica* was the first Asian oak with a chromosome-level assembly [6, 19]. However, no high-quality genomic data is available for *Q. fabri,* a species native to the subtropical regions of China.

*Quercus fabri* exhibits a leaf structure, fruit type, and tree morphology closely resembling those of *Q. mongolica*. Because of its attractive and robust wood texture along with its resistance to corrosion, it is frequently chosen as a premium material for construction and interior design purposes [20, 21]. The acorns produced by this tree are high in starch and various essential nutrients, making them suitable for processing into products like tofu and vermicelli [22]. In addition, the branches of *Q. fabri* can also be used as effective cultivation substrates for mushrooms with excellent flavor. Despite its edible value, research on *Q. fabri* has mainly focused on genetic diversity, with limited studies on its genome or functional characteristics related to mushroom culture substrate use [20].

At present, investigations into *Q. fabri* mainly focus on examining its genetic composition and diversity. In contrast, the genomic characterization and the mechanistic understanding of its role in flavor enhancement for mushroom cultivation remain unexplored [20]. Existing studies lack comprehensive genomic resources and fail to resolve the metabolic pathways underlying mushroom flavor improvement. This study aims to assemble and annotate a chromosome-level genome of *Q. fabri*, compare it with close relatives to investigate its evolutionary relationships, and investigates amino acid metabolism that may contribute to the enhanced flavor of mushrooms cultivated on *Q. fabri* substrate. It uncovers the essential mechanism of this flavor improvement for the first time.

## Results

### Genome survey and assembly

The genome of *Q. fabri* was sequenced using the DNBSEQ platform, resulting in a total sequencing output of ~58.18 Gb. After filtering, the clean reads amounted to 57.52 Gb. To estimate the genome size, an initial genome survey was performed utilizing the 57.52 Gb of clean reads. K-mer analysis revealed that the final adjusted genome size is roughly 755.32 Mb, displaying a heterozygosity rate of 1.93% alongside a duplication rate of 52.03% (Fig. S1). This suggests that the genome exhibits both significant heterozygosity and a considerable duplication rate.

This project developed two libraries of 15 Kb each and conducted HiFi sequencing on them. Sequencing was carried out on a total of two cells, generating a raw data volume of 989.36 Gb. Following the processing of HiFi data, 3 725 025 valid reads were acquired, amounting to 63.01 Gb. Based on the survey’s conclusion that the genome size is roughly 755.32 Mb, the valid reads of 63.01 Gb are estimated to provide ~82× coverage.

The initial assembly of HiFi sequencing outcomes produced a genome size of 836.696 Mb along with a contig N50 measuring 61.8 MB. A Benchmarking Universal Single-Copy Orthologs (BUSCO) integrity assessment was carried out on these preliminary assembly findings, yielding a BUSCO value of 98.7%. Approximately 133.39 Gb of Hi-C data (Q20 > 97%) was generated. Following modifications, the final assembly yielded an approximate size of 836.74 Mb, and a subsequent BUSCO integrity evaluation revealed a BUSCO value of 98.8% (Table S1). The genome sequence, measuring 816.69 Mb in length, was located on 12 chromosomes, representing roughly 97.6% (Fig. 1, Fig. S2, and Table S2).

### Genome annotation

Approximately 398.87 Mb of repetitive sequences, which make up ~47.67% of the entire genome, were identified through the use of both homology-based and *de novo* prediction techniques. Among these repetitive sequences, ~77 105 694 bp, accounting for roughly 9.22% of the genome, were categorized as tandem repeats. Additionally, 73 504 704 bp (8.78% of the genome) and 63 867 553 bp (7.63% of the genome) were identified as transposable elements with the help of RepeatMasker and RepeatProteinMask for genomic sequence annotation, respectively. Furthermore, 354 446 473 bp, representing 42.36% of the genome, were classified as transposable elements through *de novo* prediction methods.

**Figure 2:**
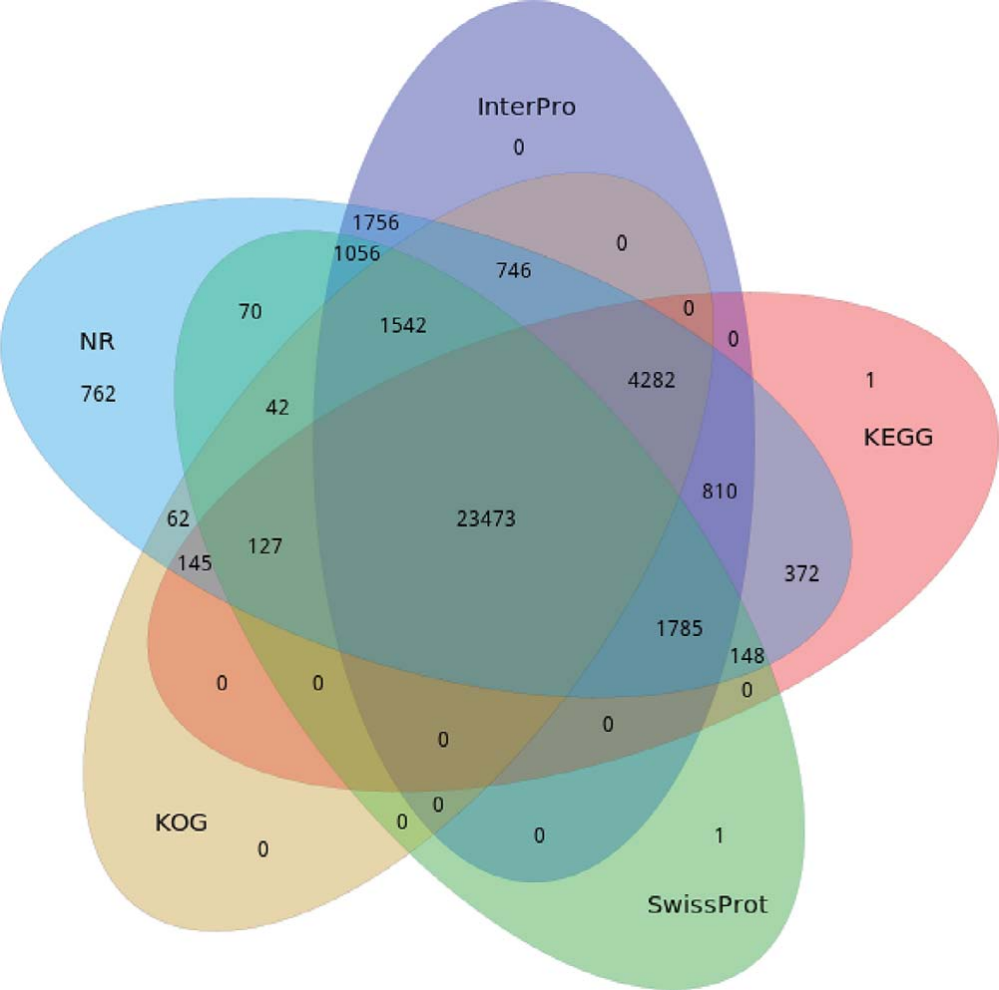
Venn diagram of the number of functional annotation results of *Q. fabri* genes in five databases: NR, InterPro, KEGG, SwissProt, and KOG.

Using homology-based prediction methods, we identified and annotated a total of 37 182 protein-coding genes (Table S3). Of these, the Nr database provided annotations for the largest number, tallying 37 178, which represents 99.94% of the overall predicted gene count. The overlap of functional annotations across the five databases is illustrated in Fig. 2, revealing that 23 473 genes received functional annotations in all five sources. The BUSCO analysis indicated that the annotated genome encompassed 1609 conserved proteins, yielding a completeness score of 99.69%. Out of these, 1533 (94.98%) were identified as single-copy genes, while 76 (4.71%) were classified as multiple copies, indicating a strong level of completeness in the annotation of protein-coding genes.

### Genome evolution

The clustering of the protein sequence alignment results for *Q. fabri* and various other species—namely, *Arabidopsis thaliana*, *Castanea mollissima*, *Juglans regia*, *Populus trichocarpa*, *Q. acutissima*, *Q. lobata*, *Q. robur*, *Solanum lycopersicum*, *Carya illinoinensis*, *Fagus sylvatica*, *Oryza sativa*, *Punica granatum*, *Q. mongolica*, *Q. suber*, and *Vitis vinifera*—was accomplished using OrthoFinder. This analysis revealed pertinent gene family information, with a total of 36 908 annotated genes (accounting for 99.21% of all genes) from *Q. fabri* being categorized into 16 773 distinct gene families (Table S4).

**Figure 3:**
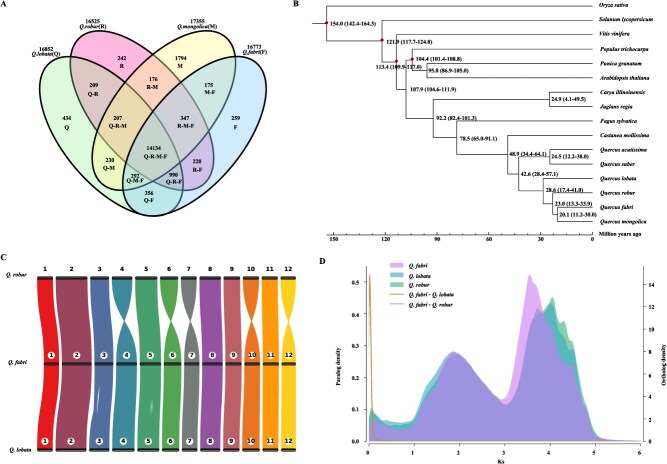
Evolutionary analysis of the *Q. fabri* genome. **A**. Venn diagram of the number of homologous gene families between *Q. fabri* and three closely related oak trees (*Q. lobata*, *Q. robur*, and *Q. mongolica*). **B**. Estimated divergence time of *Q. fabri* and other species. The numbers on the branches indicate the estimated divergence time (million years ago), and the divergence time at the dot indicates calibration time points. **C**. Chromosome collinearity diagram between *Q. fabri* and *Q. lobata* and *Q. robur* genomes. **D**. Ks distribution diagram. The X-axis is the Ks value, and the Y-axis represents the density of paralog and ortholog gene pairs, respectively.

An analysis of the homologous gene families of *Q. fabri* alongside three related species (*Q. mongolica*, *Q. robur*, *Q. lobata*) revealed a total of 14 134 homologous gene families shared among the four *Quercus* species (Fig. 3A). The phylogenetic tree suggested a close relationship between *Q. fabri* and *Q. mongolica*, indicating that these two oak species likely shared a common ancestor ~20.1 million years ago. Furthermore, the estimated divergence dates indicate that *Q. fabri* separated from *Q. robur* ~23.0 million years ago, while its divergence from *Q. lobata* occurred ~28.6 million years ago (Fig. 3B).

The relationships of synteny among the 12 chromosomes of *Q. fabri*, *Q. robur*, and *Q. lobata* were analyzed in greater depth. Syntenic blocks were created through a comparative analysis of the *Q. fabri* genome alongside those of *Q. robur* and *Q. lobata* (Fig. 3C, Table S5). The findings demonstrated a close genetic relationship among *Q. fabri*, *Q. robur*, and *Q. lobata*, suggesting that the genomes of these various oak species exhibit a significant degree of conservation.

The distribution map of synonymous substitution rates (Ks) for paralogous gene pairs reveals that *Q. fabri*, *Q. robur*, and *Q. lobata* exhibit two prominent peaks. For *Q. fabri*, these peaks are situated at Ks values of 1.91 and 3.54, corresponding to the two most recent whole-genome duplication (WGD) events that took place. Examining the Ks distribution for orthologous gene pairs shows that the peak Ks values for both *Q. fabri* and *Q. lobata*, as well as for *Q. fabri* and *Q. robur*, are all found at a Ks value of 0.01. This finding suggests that their divergence occurred in a very recent timeframe (Fig. 3D).

### Expansion and contraction of gene families

**Figure 4:**
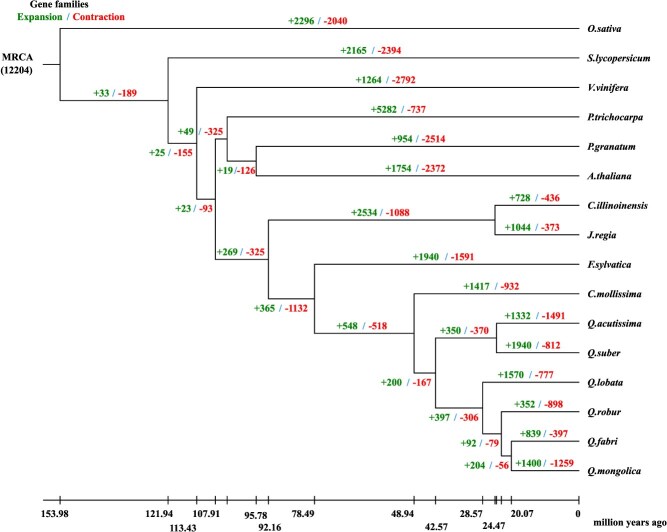
Statistical diagram of expansion and contraction of gene families in *Q. fabri* and other species. The left numbers represent the number of gene families that have expanded during the evolution of the species, and the right numbers represent the number of gene families that have contracted during the evolution of the species. MRCA，Most Recent Common Ancestor.

Upon conducting an analysis of gene family expansion and contraction, it was observed that a total of 839 gene families experienced expansion while 397 families exhibited contraction within the 16 773 gene families of *Q. fabri* (Fig. 4). Notably, the count of gene families that showed significant expansion and contraction were 303 and 8, respectively, with associated gene counts of 4733 and 119. Based on the results from gene ontology (GO) annotation, these genes were categorized functionally, and the data indicated a notable enrichment of the expanded genes in 129 GO terms, whereas the contracted genes showed significant enrichment in 19 GO terms (Table S6, Table S7). Among the GO terms that experienced significant expansion, the leading three were identified as RNA–DNA hybrid ribonuclease activity, nucleic acid binding, and NADH (Nicotinamide adenine dinucleotide) dehydrogenase (ubiquinone) activity; conversely, the top three GO terms with significant contraction comprised xylan 1,4-beta-xylosidase activity, xylan catabolic process, and DNA integration.

Utilizing the available KEGG annotation data, a functional enrichment analysis of KEGG was conducted, revealing that the expanded gene set showed significant enrichment in 114 KEGG pathways. Conversely, the contracted gene set displayed significant enrichment in only 22 KEGG pathways (Table S8, Table S9). Among the top 20 KEGG pathways that were notably expanded, a variety of pathways associated with amino acid metabolism were identified, including those for cyanoamino acid metabolism, beta-alanine metabolism, phenylalanine metabolism, as well as those for glycine, serine, threonine metabolism, and tyrosine metabolism. The increase in these gene families may have contributed to a broader diversity and elevated levels of amino acids in *Q. fabri*. In the contracted KEGG pathways, the three most significant ones were related to the sesquiterpenoid and triterpenoid biosynthesis, base excision repair, and mRNA surveillance pathway.

### *Q. fabri* has a richer content of amino acid substances

**Figure 5:**
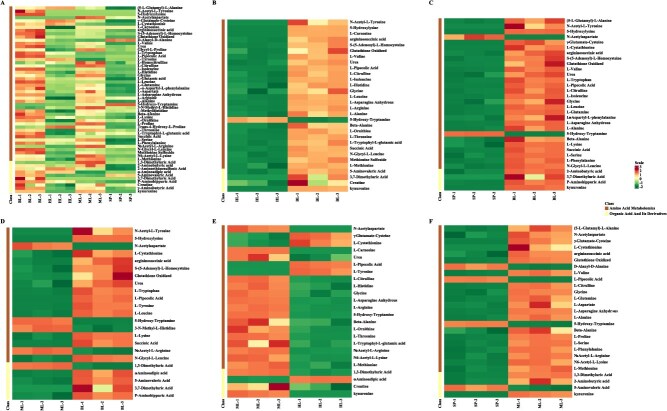
Heatmaps of clustering of differential amino acid metabolites among *Q. fabri*, *Q. aliena*, *Q. acutissima*, and *Q. variabilis*. **A**. Heatmap of clustering of differential amino acid metabolites among four oak species. **B**. Heatmap of clustering of differential amino acid metabolites between *Q. aliena* and *Q. fabri*. **C**. Heatmap of clustering of differential amino acid metabolites between *Q. variabilis* and *Q. fabri*. **D**. Heatmap of clustering of differential amino acid metabolites between *Q. acutissima* and *Q. fabri*. **E**. Heatmap of clustering of differential amino acid metabolites between *Q. acutissima* and *Q. aliena*. **F**. Heatmap of clustering of differential amino acid metabolites between *Q. variabilis* and *Q. acutissima*. Three biological replicates were analyzed for each species. BL, *Q. fabri*; HL, *Q. aliena*; ML, *Q. acutissima*; SP, *Q. variabilis*.

By conducting an analysis and comparative study of the amino acid metabolism profiles among *Q. fabri* and three related oak species—*Q. aliena*, *Q. acutissima*, and *Q. variabilis*—the findings revealed that *Q. fabri* typically demonstrated elevated levels of amino acids in contrast to *Q. aliena* and *Q. variabilis* (Fig. 5A–C, Figs S3 and S4). Moreover, a comparison between *Q. fabri* and *Q. acutissima* indicated that the majority of the identified amino acids in *Q. fabri* were present at greater concentrations than in *Q. acutissima* (Fig. 5D, Fig. S5). Additionally, when assessing *Q. acutissima* against *Q. aliena* and *Q. variabilis*, a higher concentration of most detected amino acids was observed in *Q. acutissima* compared to the other two oak species (Fig. 5E and F, Figs S6 and S7).

**Figure 6:**
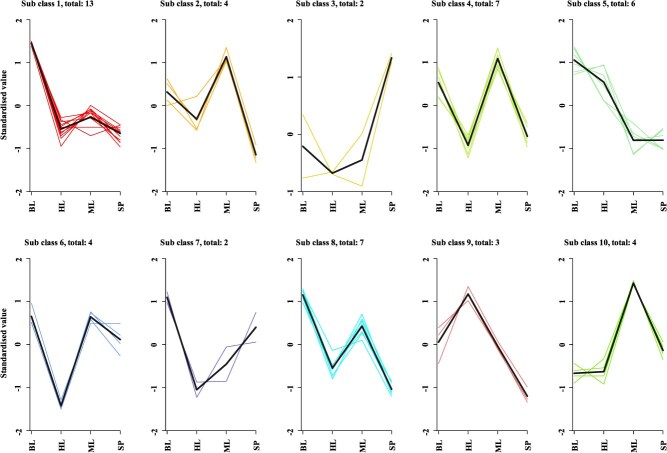
K-means clustering analysis of differential amino acid metabolites in *Q. fabri*, *Q. aliena*, *Q. acutissima*, and *Q. variabilis*. BL, *Q. fabri*; HL, *Q. aliena*; ML, *Q. acutissima*; SP, *Q. variabilis*.

**Figure 7:**
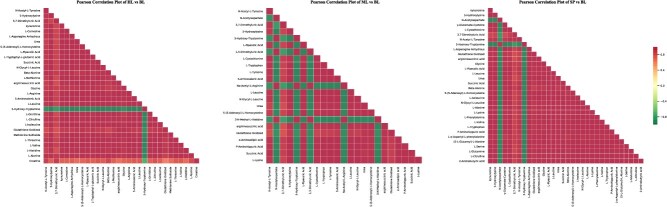
Heatmaps of correlation analysis of differential amino acid metabolites between different oak trees and *Q. fabri*. BL, *Q. fabri*; HL, *Q. aliena*; ML, *Q. acutissima*; SP, *Q. variabilis*.

To explore the variations in amino acid content across various oak tree species, we performed K-means clustering analysis subsequent to the standardization and centering of the amino acid concentrations. The findings uniformly demonstrated that, in the majority of subgroups, *Q. fabri* exhibited a greater amino acid content compared to the remaining three oak species (Fig. 6, Table S10). By conducting a correlation analysis on the amino acids that showed significant differences in their contents, we noted that the positive correlations among many of these amino acids were notably robust (Fig. 7). This observation indicated a strong metabolic relationship or potential regulatory interaction among them.

**Figure 8:**
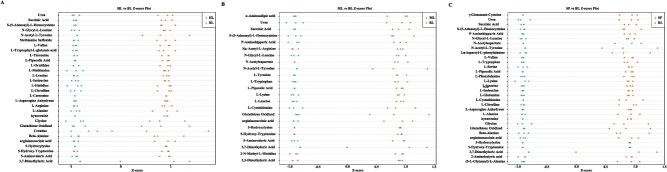
*Z*-scores diagrams of differential amino acid metabolites between different oak trees and *Q. fabri*. The X-axis represents the *Z-*value, and the Y-axis represents differential amino acid metabolites. The *Z*-value is normalized from the differential metabolites in different samples. Points of different colors represent different groups of samples. BL, *Q. fabri*; HL, *Q. aliena*; ML, *Q. acutissima*; SP, *Q. variabilis*.

The six amino acids celebrated for their distinct umami tastes—glutamic acid, aspartic acid, phenylalanine, alanine, glycine, and tyrosine—are commonly termed flavor amino acids. In our investigation, we conducted a comparison and analysis of the flavor amino acid content in *Q. fabri*, *Q. aliena*, *Q. acutissima*, and *Q. variabilis*. The findings revealed that the amounts of glutamic acid, aspartic acid, alanine, glycine, tyrosine, and their derivatives in *Q. fabri* were markedly higher than those in *Q. aliena* (Fig. 8A, Table S11). Moreover, *Q. fabri* showed significantly increased levels of tyrosine and its derivatives when compared to *Q. acutissima* (Fig. 8B, Table S12). Additionally, when assessing *Q. fabri* alongside *Q. variabilis*, it was found to have significantly elevated levels of glutamic acid, phenylalanine, alanine, glycine, tyrosine, and their derivatives (Fig. 8C, Table S13). These findings may help elucidate why mushrooms grown from *Q. fabri* branches have a richer flavor profile.

**Figure 9:**
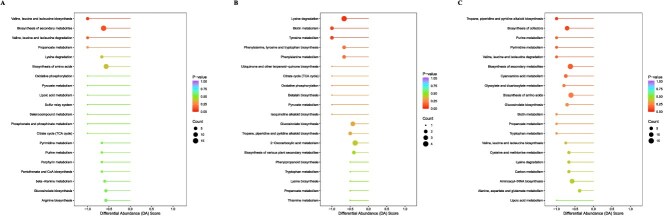
Differential Abundance Score (DA Score) diagrams of differential metabolic pathways between different oak trees and *Q. fabri*. **A**. DA Score diagrams of differential metabolic pathways between *Q. aliena* and *Q. fabri*. **B**. DA Score diagrams of differential metabolic pathways between *Q. acutissima* and *Q. fabri*. **C**. DA Score diagrams of differential metabolic pathways between *Q. variabilis* and *Q. fabri*. The X-axis represents the DA Score, and the Y-axis represents the name of the differential metabolic pathway. DA Score reflects the overall changes in all metabolites in metabolic pathways. A score of 1 indicates that the expression trend of all identified metabolites in this pathway is upregulated, and −1 indicates that the expression trend of all identified metabolites in this pathway is downregulated. The length of the line segment represents the absolute value of DA Score, and the size of the dots at the endpoints of the line segment represents the number of differential metabolites in the pathway. The dot reflects the size of the *P-*value.

The KEGG analysis investigating the overall alterations in amino acid metabolic pathways with different contents indicated that the pathways in three oak species—*Q. aliena*, *Q. acutissima*, and *Q. variabilis*—demonstrated substantial downregulation when compared to *Q. fabri*. This encompassed several biosynthetic and metabolic pathways for amino acids. For example, a comparison of *Q. aliena* with *Q. fabri* highlighted a notable downregulation in pathways related to the synthesis of amino acids, including valine, leucine, and isoleucine, as well as arginine synthesis (Fig. 9A). Similarly, the pathways for tryptophan metabolism and lysine biosynthesis exhibited significant downregulation when *Q. acutissima* was compared to *Q. fabri* (Fig. 9B). Moreover, a comparison of *Q. variabilis* with *Q. fabri* revealed considerable downregulation in amino acid metabolism pathways, which included the biosynthesis of amino acids, valine, leucine, isoleucine, as well as cysteine and methionine metabolism (Fig. 9C). Additionally, there was significant downregulation in metabolic pathways associated with flavor amino acids, comprising beta-alanine metabolism, tyrosine metabolism, and the biosynthesis and metabolism of phenylalanine, tryptophan, alanine, aspartate, and glutamate (Fig. 9A–C).

### Mushrooms cultured from *Q. fabri* contain more amino acids

**Figure 10:**
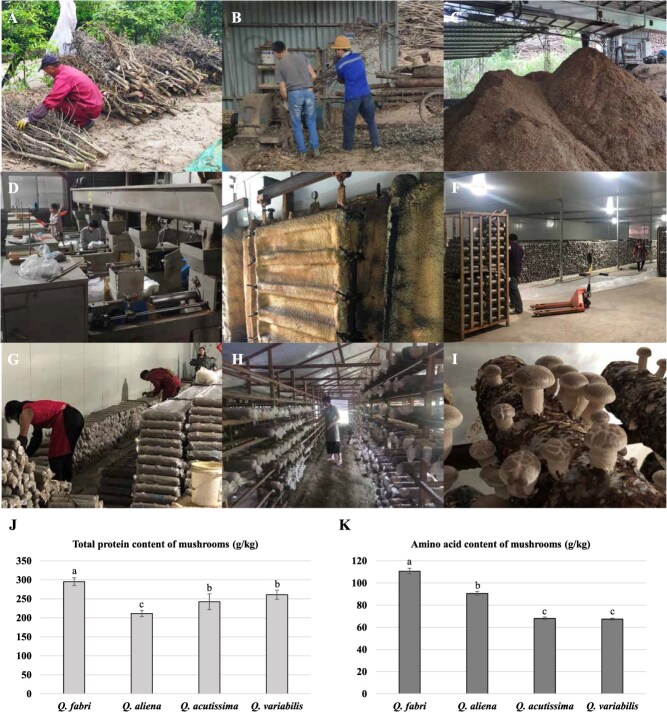
Comparison of total protein content and amino acid content in mushrooms cultured from different oak trees. **A**. Classification of different oak trees to be used as mushroom culture substrates. **B**. Oak trees were crushed by machines. **C**. Display of crushed oak wood samples. **D**. Bagging oak sawdust. **E**. Sterilization of bagged oak substrate. **F**. Bagged oak substrate cooling. **G**. Inoculate with bagged oak substrate. **H**. Bagged oak substrate shelf management. **I**. Mushroom growth and harvesting. **J**. Comparison of total protein content in mushrooms cultured from different oak trees. **K**. Comparison of amino acid content in mushrooms cultured from different oak trees. Data are the mean values. Error bars represent the standard deviation of 60 biological replicates. Different letters indicate significant differences (*P* < 0.05).

Based on the comparative results of the amino acid metabolism profiles of the four types of oak trees mentioned above, we utilized these oak trees as culture substrates to conduct experiments on mushroom cultivation (Fig. 10A–I), as well as total protein, and amino acid detection in the mushrooms (Fig. 10J–K). The experimental results indicated that the total protein content of mushrooms cultivated using *Q. fabri* as the culture substrate was the highest, reaching 295.3 g/kg. In contrast, the total protein content of mushrooms cultivated using *Q. variabilis* ranked second, at ~261.0 g/kg. The total protein content of mushrooms cultured from *Q. acutissima* was third, at ~242.4 g/kg, while the total protein content of mushrooms cultured from *Q. aliena* was the lowest, at only 211.6 g/kg (Fig. 10J). Additionally, the amino acid content in mushrooms cultivated using *Q. fabri* was the highest, reaching 110.75 g/kg. The amino acid content in mushrooms cultivated using *Q. aliena* ranked second, at ~90.66 g/kg, followed by mushrooms cultivated using *Q. acutissima*, which has an amino acid content of ~68.10 g/kg. Finally, the amino acid content of mushrooms cultured using *Q. variabilis* is the lowest, at only 67.44 g/kg (Fig. 10K). These results demonstrated that the total protein and amino acid content of mushrooms cultivated using *Q. fabri* sawdust as a substrate were significantly higher than those of the other three oak trees, indicating that they were richer in nutrients and more flavorful.

## Discussion

There are ~500 species of *Quercus* worldwide, encompassing both evergreen and deciduous tree species. This genus is the largest within the Fagaceae family and is distributed spanning 46 degrees of latitude from north to south and 75 degrees of longitude from east to west [23]. In China alone, there are >60 species of *Quercus*, representing one-eighth of the global total, with a wide distribution from northern temperate zones to tropical mountains [24]. Notably, evergreen oak forests are predominantly found in the Qinling Mountains, south of the Huaihe River, as well as in tropical and subtropical regions. China serves as one of the primary centers and origins for *Quercus* [25]. Many species are foundational and dominant within warm temperate and temperate forests, possessing significant social, ecological, and economic value. Beyond their common use as timber for construction, vehicles, boats, and furniture, *Quercus* species also feature bark-bearing trunks or branches that serve as excellent materials for cultivating edible fungi [9]. These fungi often thrive on standing or fallen oak trees, leading to a tradition of mushroom harvesting in oak forests. Concurrently, there is substantial market demand for the use of oak branches as raw materials in the artificial cultivation of edible fungi, such as shiitake mushrooms and other varieties [26].

Shiitake mushrooms belong to the Basidiomycetes class, Agaricales order, Tricholomataceae family, and the *Lentinus* genus. They originated in China and represent the second largest mushroom species globally [27] . Renowned for their delicious flavor and rich aroma, shiitake mushrooms are also packed with nutrients and exhibit significant medicinal and nourishing properties [28, 29]. The amino acid content in shiitake mushrooms is exceptionally rich. Of the 20 types of amino acids that constitute proteins, shiitake mushrooms contain 18. Notably, these include glutamic acid, aspartic acid, phenylalanine, alanine, glycine, and tyrosine, and these six flavor amino acids contribute to the distinctive umami flavor characteristic of shiitake mushrooms [30].

Gan *et al.* conducted mushroom cultivation experiments using five species of oak trees and several other common broad-leaved tree species as substrates [31]. They compared the yield, amino acid content, and polysaccharide content of the mushrooms. The results indicated that both the yield and amino acid content of mushrooms cultivated on oak trees were significantly higher than those grown on other broad-leaved tree species. Additionally, the growth cycle of shiitake mushrooms grown on oak wood is shorter than that of those grown on other types of wood, completing within 20–30 days, which allows for faster and more efficient harvesting [32]. Notably, mushrooms grown on oak exhibit a more desirable taste compared to those cultivated on other woods. Li *et al*. selected nine common oak tree varieties to cultivate shiitake mushrooms and analyzed their yield and nutritional content [9]. The study concluded that *Q. fabri* and *Castanopsis tibetana* are particularly suitable as high-yield cultivation substrates for shiitake mushrooms. However, there are relatively few studies investigating why oak trees, specifically *Q. fabri*, cultivate mushrooms with a superior taste. To address these questions, it is essential to explore the metabolic profile of *Q. fabri*. Additionally, a more in-depth examination of the genetic factors contributing to this phenomenon necessitates a comprehensive understanding of the genomic information associated with *Q. fabri*.

In this study, we generated a high-quality chromosome-scale assembly of the genome of *Q*. *fabri* using a combination of PacBio Sequel II, DNBseq™ and Hi-C sequencing platforms. This *Q*. *fabri* genome exhibits exceptional continuity and chromosome-level completeness, with contig and scaffold N50 values of 61.8 and 69.2 Mb, respectively. Notably, the contig N50 of *Q. fabri* is substantially longer than those of closely related oak species including *Q. variabilis*, *Q. aliena*, and *Quercus dentata*, while the scaffold N50 is similar to the published value of 66.74 Mb for *Q. mongolica* [18, 19]. The assembled genome was ~836.74 Mb in length, and about 97.6% of the assembled genome sequences could be anchored to 12 chromosomes (Fig. 1, Table S2). The anchoring rate is superior to that of *Q. mongolica* (95.65%) or *Q. robur* (96.00%) [6, 19]. These results establish *Q. fabri* genome as a superior reference resource for Fagaceae genomic studies. Through evolutionary analysis, the results indicated that phylogenetic trees based on single-copy orthologous genes revealed that *Q*. *fabri* and *Q*. *mongolica* diverged relatively recently, while *Q*. *robur* diverged from the ancestor of these two species much earlier (Fig. 3B). This conclusion may also explain the similarities in leaf morphology, male and female flower morphology, fruit morphology, tree body morphology, and the flowering and fruiting stages of *Q*. *fabri* and *Q*. *mongolica*. Additionally, through the analysis of pollen, leaf epidermal morphology, anatomical characteristics, and DNA, *Quercus* species distributed in China can be categorized into five distinct characterization Sections: Section *Quercus*, Section *Aegilops*, Section *Engleriana*, Section *Brachylepides*, and Section *Echinolepides* [25, 33]. Most existing phylogenetic studies on *Quercus* tree species focus on three Sections: Section *Quercus*, Section *Aegilops*, and Section *Brachylepides* [19, 34–36]. Among these, *Q*. *acutissima*, *Q*. *variabilis*, and *Quercus chenii* collectively form the Section *Aegilops* within the Chinese genus *Quercus*. The Section *Quercus* primarily comprises *Q*. *fabri*, *Q. aliena*, *Q*. *mongolica*, *Q*. *liaotungensis*, and *Q*. *dentata*. The evolutionary analysis results in this study also revealed that among the *Quercus* tree species, *Q*. *acutissima* and *Q*. *variabilis*, two tree species in the Section *Aegilops*, diverged from *Q*. *fabri* the earliest, ~42.6 million years ago (Fig. 3B).

By analyzing the genome family expansion of *Q*. *fabri*, we discovered the evolutionary reorganization of gene pathways, leading to functional optimization and reduced genetic redundancy. GO annotation revealed that genes related to RNA–DNA hybrid ribonuclease activity contribute to organellar genome stability, while those involved in NADH dehydrogenase activity participate in mitochondrial electron transport, both potentially enhancing the environmental adaptability of *Q. fabri* (Table S6) [37, 38]. KEGG pathway analysis showed that several expanded pathways are linked to critical physiological functions: cyanoamino acid metabolism is widely recognized for enhancing plant resilience to abiotic stresses and pathogen attack; β-alanine metabolism supports lignin biosynthesis and cellular homeostasis; both phenylalanine and tyrosine metabolism facilitate the accumulation of aromatic secondary metabolites and volatile fragrance compounds; and glycine, serine, and threonine metabolism are essential for carbon and nitrogen assimilation [39–43]. As amino acid metabolism plays a fundamental role in plant growth, development, and environmental adaptation, the expansion of these pathways likely enhances the metabolic flexibility of *Q. fabri*. Furthermore, the interconversion of amino acids—such as the synthesis of glutamic acid from ornithine and proline from arginine—illustrates the biochemical versatility supported by these expanded metabolic networks. Collectively, these expansions suggest that amino acid metabolism has been a key driver of *Q. fabri*’s adaptive evolution, and may contribute to a greater diversity and higher contents of amino acids in this species (Table S8).

To further verify this conclusion phenotypically, we selected *Q. aliena*, which belongs to the same Section as *Q*. *fabri*, along with *Q*. *acutissima* and *Q*. *variabilis*, both of which belong to the Section *Aegilops* that emerged earlier in the divergence time of *Q*. *fabri* to perform amino acid metabolism profile detection. The results aligned with our expectations. The contents of most amino acids in *Q*. *fabri* were significantly higher than those in the other three oak species (Fig. 5, Fig. 6, Fig. S3, Fig. S4), especially phenylalanine, tyrosine, tryptophan, leucine, and isoleucine, which are key precursors involved in the biosynthesis of volatile aromatic compounds contributing to aroma. Furthermore, the corresponding investigation and analysis of the differential amino acid KEGG metabolic pathways revealed that, compared to the other three oak trees, a substantial number of amino acid biosynthesis and metabolic pathways in *Q*. *fabri* exhibited an upregulation trend, particularly in pathways associated with flavor amino acids (Fig. 9). We have also conducted detailed analysis on the metabolic pathways of certain specific amino acids. For instance, valine, leucine, and isoleucine, classified as branched-chain amino acids (BCAAs) due to their short-branched side chains, are synthesized through pathways in which branched-chain aminotransferase (BCAT) catalyzes the final transamination step. The catabolism of BCAAs in plants occurs primarily in mitochondria, where enzymes such as branched-chain keto acid dehydrogenase (BCKDH) convert α-keto acids to acyl-CoA derivatives, which are further oxidized through β-oxidation-like pathways. Phenylalanine, tyrosine, and tryptophan, as aromatic amino acids, serve as important precursors for the phenylpropanoid pathway and indole glucosinolate biosynthesis, with phenylalanine ammonia-lyase (PAL) and tryptophan decarboxylase (TDC) acting as key enzymes in these respective pathways. These pathways produce secondary metabolites such as lignin, flavonoids, and indole derivatives [41, 44, 45]. In addition, through comparative analysis, we found that the contents of several flavor amino acids and their derivatives in *Q*. *fabri* were significantly higher than those in the other three oak species (Fig. 8, Fig. S3, Fig. S4). However, do mushrooms cultivated on *Q*. *fabri* as a substrate also exhibit enhanced amino acid content? Subsequent experiments involving mushroom cultivation and amino acid detection further corroborated this conclusion (Fig. 10).

In summary, this study represents the first comprehensive whole-genome sequencing and assembly of *Q*. *fabri*, a species within the *Quercus* genus that is distributed across the subtropical regions of China. Evolutionary analysis revealed significant gene expansion in numerous gene families associated with amino acid metabolism within the genome. Subsequently, amino acid testing was performed on *Q*. *fabri*, other related *Quercus* tree species, and mushrooms cultivated using these trees as substrate materials. The results also indicated that the types and contents of amino acids, particularly those associated with flavor production and their derivatives, in *Q*. *fabri* and mushrooms cultured from *Q*. *fabri*, were significantly more than those found in other *Quercus* species. These experimental analysis results elucidate why utilizing *Q*. *fabri* as a substrate for mushroom cultivation can enhance the flavor profile of the cultured mushrooms.

## Materials and methods

### Plant material, sample preparation, and extraction

The *Quercus* tree species utilized in this study were all planted in the experimental forest farm of the Research Institute of Subtropical Forestry of Chinese Academy of Forestry, located in Hangzhou, Zhejiang Province, China (Fig. 1). Fresh leaves were collected from 5-year-old *Q*. *fabri* for whole-genome sequencing. Additionally, branches were gathered from 5-year-old *Q*. *fabri*, as well as from *Q. aliena*, *Q. acutissima*, and *Q. variabilis*, to detect amino acid metabolic profile. Following collection, the fresh samples were immediately frozen using liquid nitrogen and stored at −80°C for subsequent investigations. A modified cetyltrimethylammonium bromide (CTAB) protocol was employed to extract genomic DNA from the leaves. To assess the quality and concentration of the extracted genomic DNA, a NanoDrop 2000 spectrophotometer and a Qubit 2.0 fluorometer were utilized. Additionally, the integrity of the DNA was confirmed via electrophoresis on a 0.8% agarose gel. For the analysis of the amino acid metabolic profile, branches from the Quercus tree species were thawed and crushed; specifically, 0.05 g of the sample was combined with 500 μl of a 70% methanol/water solution. The mixture was vortexed for 3 min at a speed of 2500 r/min and subsequently centrifuged at 12 000 r/min for 10 min at 4°C. A volume of 300 μl of supernatant was then transferred to a new centrifuge tube and stored at −20°C for 30 min. Afterward, the supernatant underwent another centrifugation at 12 000 r/min for 10 min at 4°C. Following this centrifugation step, 200 μl of the supernatant was extracted for further analysis utilizing a Protein Precipitation Plate in conjunction with LC–MS.

### Genome sequencing

In the process of extracting DNA from the whole genome of *Q. fabri*, part of the samples underwent fragmentation using the Megaruptor system, resulting in the isolation and precise recovery of 15-kb fragments with the help of Sage ELF. This procedure created a PacBio HiFi library characterized by high fragment size consistency. Meanwhile, another segment of the samples was randomly fragmented using the Covaris ultrasonic high-performance sample processing system, leading to the generation of a short-fragment library with an approximate size of 350 bp. Libraries featuring long insert sizes of ~15 kb and short insert sizes ~350 bp were constructed and sequenced in accordance with standard protocols on the MGISEQ-2000 platform and the Pacbio Sequel II platform, respectively. In the preparation of a Hi-C library, genomic DNAs were fixed using formaldehyde and subjected to shearing with a restriction enzyme (MboI). The resulting library was sequenced on the MGISEQ-2000 platform. DNBSEQ clean data was processed to filter out adaptors and low-quality reads using SOAPnuke v2.1.0, employing the parameters ‘-n 0.02 -l 20 -q 0.4 -i -G 2 --polyX 50 -Q 2 --seqType 0’.

### Genome size estimation and genome assembly

Prior to assembling the genome, the 21 k-mer from DNBSEQ clean reads was analyzed using Jellyfish v2.2.6 with its default settings. GenomeScope was employed to estimate genome size, heterozygosity levels, and repeat content.

Initially, HiFi clean reads were utilized to assemble contigs via Hifiasm v0.15.4 with standard parameters, followed by the detection and elimination of redundant contigs with Purge_haplotigs v1.1.1. Subsequently, Hi-C clean reads were aligned to the draft genome assembly, and interaction matrices were generated using Juicer v1.5.6. The 3D-DNA pipeline was then applied to anchor primary contigs into a chromosome-level assembly based on the output from the Juicer pipeline. To evaluate the completeness and quality of the genome assembly, the BUSCO evaluation score was employed with the Embryophyta_odb10 database.

### Genome annotation

Repetitive elements were detected using a mix of homolog-based and *de novo* strategies. To find repeats, RepeatMasker and RepeatProteinMask were used to align against the Repbase database. For creating a *de novo* TE library, LTR-FINDER and RepeatModeler were utilized to establish consensus sequences of transposable elements (TEs), while tandem repetitive sequences were obtained using TRF. Subsequently, RepeatMasker was applied to uncover and classify repetitive sequences through the merged library of the *de novo* TEs.

RNA libraries with an insert size ranging from 300 to 400 bp were generated following the guidelines provided by the manufacturer for the DNBSEQ sequencing platform, and paired-end sequencing was conducted on the MGISEQ-2000 platform. The transcriptome reads were mapped onto the assembled genome sequence employing HISAT2, after which GeMoMa was utilized to gather intron information from all samples for further auxiliary annotation. For gene structure prediction, the gene sets and genome sequences of five closely related species (*C. illinoinensis*，*J. regia*，*Q. lobata*，*Q. robur*，*Q. suber*) were incorporated using GeMoMa v1.9. The five predicted gene sets were merged through GeMoMa, and instances of alternative splicing were eliminated to derive the final gene set. To evaluate the completeness of the gene models, the gene content was consulted against the embryophyta_odb10 database using BUSCO.

Gene functional annotation was conducted by utilizing the consensus of both sequence and domain information. Protein sequences were aligned against NR, KEGG, SwissProt, TrEMBL, and KOG databases using BLAST v2.2.26 with the settings ‘-p blastp -e 1e-05’. The domains were identified and predicted utilizing InterProScan in conjunction with the publicly accessible InterPro database. GO terms associated with each gene were inferred from the InterPro annotations.

### Genome evolution analysis

OrthoFinder v 2.3.11 was utilized to identify orthologous and paralogous genes from *Q. fabri* and various other species, including *A. thaliana*, *C. mollissima*, *J. regia*, *P. trichocarpa*, *Q. acutissima*, *Q. lobata*, *Q. robur*, *S. lycopersicum*, *C. illinoinensis*, *F. sylvatica*, *O. sativa*, *P. granatum*, *Q. mongolica*, *Q. suber*, and *V. vinifera*. For the phylogenetic tree construction, we first conducted multiple sequence alignment of all single-copy orthologous gene families, subsequently concatenating the alignment results for each species to create the phylogenetic tree. In this research, we employed 476 protein sequences from single-copy orthologous genes gathered through gene family clustering, and the phylogenetic tree was built using RAxML software. *Oryza sativa* served as the outgroup, and the arrangement of the other species in the phylogenetic tree aligned with their positions in the APG IV phylogenetic framework.

The MCMCtree tool within PAML was utilized to determine the divergence time among 16 species depicted in the phylogenetic tree. References were established using four calibration time points from the TimeTree database (http://www.timetree.org), which included the following: the divergence of *A. thaliana* and *O. sativa* (spanning 142.4–164.3 million years ago), the separation of *P. trichocarpa* from *A. thaliana* (ranging from 101.4 to 108.8 million years ago), the divergence between *A. thaliana* and *S. lycopersicum* (117.7–124.8 million years ago), and finally, the divergence of *A. thaliana* from *V. vinifera* (109.9–117.0 million years ago).

The relationship of synteny among the genomes of the three oak species (*Q. fabri*, *Q. robur*, and *Q. lobata*) is established through an all-vs-all BLASTP v2.2.30 alignment of their protein sequences, applying an e-value threshold of 1e-10. Subsequently, syntenic regions are identified using MCScanX v1.5.2 with parameters (−s 5 -e 1e-05 -a), followed by a statistical analysis. Construction of the genomic syntenic map is accomplished with the JCVI v1.1.22 software, which searches for syntenic blocks while utilizing parameters (−cscore = 0.7, https://github.com/tanghaibao/jcvi/wiki/MCscan-(Python-version)).

The rate of synonymous substitutions (Ks) can serve as a method to assess whether a species has experienced WGD events throughout its evolutionary timeline, as well as to differentiate the relative timings of these duplications by comparing them to the divergence times of other species. To compute the Ks value, we initially aligned the protein sequences from the species using BLASTP (with an e-value <1e-5) and subsequently identified syntenic blocks utilizing MCscanX v1.5.2 (−a -e 1e-5 -s 5). Drawing on the findings from the syntenic block search, we employed the yn00 program in PAML software to derive the Ks values for the paired sequences.

CAFÉ v4.2.1 (http://sourceforge.net/projects/cafehahnlab/) was utilized to explore the expansion and contraction of gene families within a maximum likelihood framework. Input files consisted of single-copy orthologous gene families along with the estimated divergence times across various species. Families with a *P*-value of <0.01 were considered to have significant expansion or contraction and were subsequently used for enrichment analysis.

### Detection of amino acid metabolic profile

The sample extracts underwent analysis utilizing an LC-ESI-MS/MS system (UPLC, ExionLC AD, https://sciex.com.cn/; MS, QTRAP® 6500+ System, https://sciex.com/). The analytical parameters were established as follows: for HPLC, the column used was an ACQUITY BEH Amide (i.d. 2.1 × 100 mm, 1.7 μm); the solvent system consisted of water with 2 mM ammonium acetate and 0.04% formic acid (A), paired with acetonitrile containing 2 mM ammonium acetate and 0.04% formic acid (B). The gradient commenced at 90% B (0–1.2 min), reduced to 60% B (at 9 min), further decreased to 40% B (10–11 min), and ultimately ramped back up to 90% B (11.01–15 min); the flow rate was maintained at 0.4 ml/min, and the temperature was set at 40°C, with an injection volume of 2 μl. The AB 6500+ QTRAP® LC–MS/MS System featured an ESI Turbo Ion-Spray interface, which functioned in both positive and negative ion modes, and was governed by Analyst 1.6 software (AB Sciex). The operational parameters for the ESI source included: turbo spray as the ion source; a source temperature of 550°C; ion spray voltage (IS) was set to 5500 V (Positive) and −4500 V (Negative); the curtain gas (CUR) was calibrated to 35.0 psi; while DP and CE adjustments for the respective MRM transitions were performed through additional optimization. A tailored set of MRM transitions was tracked for each time frame based on the amino acids eluted during those intervals. The detection of amino acids and their metabolites was conducted by MetWare (http://www.metware.cn/) utilizing the AB Sciex QTRAP® 6500 LC–MS/MS platform.

### Differential metabolites selected, KEGG annotation, and enrichment analysis

Differential metabolites of significant interest between the groups were evaluated based on the absolute Log_2_FC (fold change). The metabolites that were identified were annotated by utilizing the KEGG compound database (http://www.kegg.jp/kegg/compound/), and these annotated metabolites were subsequently linked to the KEGG Pathway database (http://www.kegg.jp/kegg/pathway.html). Pathways where notably different metabolites were mapped were then analyzed using MSEA (metabolite sets enrichment analysis), and the significance of these pathways was assessed using *P*-values from the hypergeometric test.

### Culture of mushrooms and determination of total amino acid and protein content

Branches were collected from six plants each of 5-year-old *Q. fabri*, *Q. aliena*, *Q. acutissima*, and *Q. variabilis*, dried out, and then ground into chips with the help of a machine. These wood chips were subsequently packed into 14 × 40 cm cylindrical substrate bags, and each bag of substrate was composed of 78% wood chips from the respective oak tree species, 21% wheat bran, and 1% gypsum powder, with a moisture content adjusted to 55%. Ten substrate bags were made from the branches collected from each tree. Before arranging the substrate bags, the ground was disinfected by spraying a 15% carbendazim solution. The selected strain for this research was *Lentinus edodes* ‘Qingke 212’. The mushroom cultivation experiment took place in a facility dedicated to edible fungus cultivation in Lishui City, Zhejiang Province, using a greenhouse method for mushroom growth. Samples were gathered during the peak mushroom growth phase, and the dried mushrooms’ total amino acid and protein contents were assessed. To measure the total amino acid content, proteins in the sample are hydrolyzed with hydrochloric acid to release free amino acids. These amino acids are separated through an ion exchange column, where they undergo a color reaction with ninhydrin solution, enabling quantification with a visible light spectrophotometer. The total protein content is determined by decomposing the sample’s protein under catalytic heating, where the resulting ammonia reacts with sulfuric acid to generate ammonium sulfate. Through alkaline distillation, the ammonia is released and then absorbed with boric acid, followed by titration with a standard sulfuric acid solution. The nitrogen content is computed based on the acid consumption, which is then multiplied by a conversion factor to ascertain the protein content.

## Supplementary Material

Web_Material_uhaf156

## Data Availability

The raw data of genome sequencing of *Quercus fabri* is deposited in the NCBI SRA database with BioProject ID PRJNA1189174. The data, such as the assembled genome sequence, gene function annotation, repetitive sequences, predicted CDS, protein sequences, transcriptional factor, and amino acid metabolic profile, are available at FigShare (https://doi.org/10.6084/m9.figshare.27927681).
