# Supplementary material for: Uncovering the genetic basis for enhanced mushroom flavor in Quercus fabri through genome sequencing and metabolic profiling
Source: Hortic Res. 2025 Jul 9;12(9):uhaf156. doi: 10.1093/hr/uhaf156 (PMC12372586; doi:10.1093/hr/uhaf156)
Supplement: Web_Material_uhaf156 [file web_material_uhaf156.zip › Table S1. Statistical analysis of BUSCO evaluation results for genome assembly and annotation completeness of Q. fabri.pdf]

Table S1. Statistical analysis of BUSCO evaluation results for genome assembly and annotation completeness of *Q. fabri*

Type	Number	Percentage
Complete BUSCOs (C)	1,594	98.8%
Complete and single-copy BUSCOs (S)	1,531	94.9%
Complete and duplicated BUSCOs (D)	63	3.9%
Fragmented BUSCOs (F)	13	0.8%
Missing BUSCOs (M)	7	0.4%
Total	1,614	

Complete BUSCOs (C): Number of BUSCO genes with full-length matches in the assessment; Complete and single-copy BUSCOs (S): Subset of complete BUSCOs present as a single copy; Complete and duplicated BUSCOs (D): Subset of complete BUSCOs present in more than one copy; Fragmented BUSCOs (F): BUSCO genes with only partial alignments to the reference profiles; Missing BUSCOs (M): BUSCO genes not detected in the assembled genome.
