# Supplementary material for: Uncovering the genetic basis for enhanced mushroom flavor in Quercus fabri through genome sequencing and metabolic profiling
Source: Hortic Res. 2025 Jul 9;12(9):uhaf156. doi: 10.1093/hr/uhaf156 (PMC12372586; doi:10.1093/hr/uhaf156)
Supplement: Web_Material_uhaf156 [file web_material_uhaf156.zip › Table S2. The length information of each super scaffold.pdf]

Table S2. The length information of each super scaffold.

**Super Scaffold**	**Length (bp)**
Chr01	93,828,823
Chr02	63,093,255
Chr03	100,526,505
Chr04	69,213,983
Chr05	51,078,637
Chr06	42,931,940
Chr07	55,083,006
Chr08	58,854,868
Chr09	55,812,798
Chr10	58,192,511
Chr11	76,183,151
Chr12	91,890,674
