# Supplementary material for: Uncovering the genetic basis for enhanced mushroom flavor in Quercus fabri through genome sequencing and metabolic profiling
Source: Hortic Res. 2025 Jul 9;12(9):uhaf156. doi: 10.1093/hr/uhaf156 (PMC12372586; doi:10.1093/hr/uhaf156)
Supplement: Web_Material_uhaf156 [file web_material_uhaf156.zip › Table S3. Functional annotation statistics of genes in different databases.pdf]

Table S3. Functional annotation statistics of genes in different databases.

	**Total**	**NR**	**SwissProt**	**KEGG**	**KOG**	**TrEMBL**	**InterPro**	**GO**	**Overall**
**Number**	37,202	37,178	28,244	31,143	30,419	37,088	35,450	22,051	37,182
**Percentage**	100%	99.94%	75.92%	83.71%	81.77%	99.69%	95.29%	59.27%	99.95%
