# Supplementary material for: Uncovering the genetic basis for enhanced mushroom flavor in Quercus fabri through genome sequencing and metabolic profiling
Source: Hortic Res. 2025 Jul 9;12(9):uhaf156. doi: 10.1093/hr/uhaf156 (PMC12372586; doi:10.1093/hr/uhaf156)
Supplement: Web_Material_uhaf156 [file web_material_uhaf156.zip › Table S4. Result statistics of gene family clustering.pdf]

Table S4. Result statistics of gene family clustering.

Species	Genes number	Genes in families	Family number	Unique families	Average genes per family
Q. lobata	36,608	36,163	16,852	106	2.15
Q. robur	32,065	31,831	16,525	35	1.93
Q. suber	48,071	46,002	20,848	4,379	2.21
Q. mongolica	36,553	34,610	17,355	510	1.99
C. mollissima	33,293	31,331	17,458	312	1.79
F. sylvatica	61,185	58,361	17,705	2,053	3.3
J. regia	30,567	30,158	14,780	109	2.04
C. illinoinensis	31,352	30,916	14,748	152	2.1
P. granatum	23,641	22,777	13,946	279	1.63
P. trichocarpa	42,945	37,154	15,943	943	2.33
V. vinifera	31,315	27,607	15,766	817	1.75
S. lycopersicum	35,343	30,540	15,102	1,007	2.02
A. thaliana	27,569	25,352	13,682	752	1.85
O. sativa	42,173	33,966	14,799	2,595	2.3
Q. acutissima	31,012	29,556	16,626	205	1.78
Q. fabri	37,202	36,908	16,773	31	2.2
