# Supplementary material for: Uncovering the genetic basis for enhanced mushroom flavor in Quercus fabri through genome sequencing and metabolic profiling
Source: Hortic Res. 2025 Jul 9;12(9):uhaf156. doi: 10.1093/hr/uhaf156 (PMC12372586; doi:10.1093/hr/uhaf156)
Supplement: Web_Material_uhaf156 [file web_material_uhaf156.zip › Table S5. Collinearity statistics of the Q. fabri genome itself and collinearity statistics with the genomes of two other closely related oak trees.pdf]

Table S5. Collinearity statistics of the *Q. fabri* genome itself and collinearity statistics with the genomes of two other closely related oak trees.

Species	Number of synteny blocks	Average collinear genes per block	Number of collinear genes pairs in all blocks	Mean block length in current species (bp)
Q. fabri	30,474	9	282,728	2,315,785
Q. lobata	30,207	9	282,007	2,335,031
Q. robur	26,238	9	247,851	2,568,407
Q. fabri* vs *Q. lobata	32,641	9	306,514	2,337,276/2,259,744
Q. fabri* vs *Q. robur	35,982	9	341,411	2,351,058/2,491,231
