# Supplementary figures and images for: Uncovering the genetic basis for enhanced mushroom flavor in Quercus fabri through genome sequencing and metabolic profiling
Source: Hortic Res. 2025 Jul 9;12(9):uhaf156. doi: 10.1093/hr/uhaf156 (PMC12372586; doi:10.1093/hr/uhaf156)

# GenomeScope Profile

len:755,318,898bp uniq:48%

aa:98.1% ab:1.93%

kcov:30 err:0.465% dup:1.06 k:21 p:2

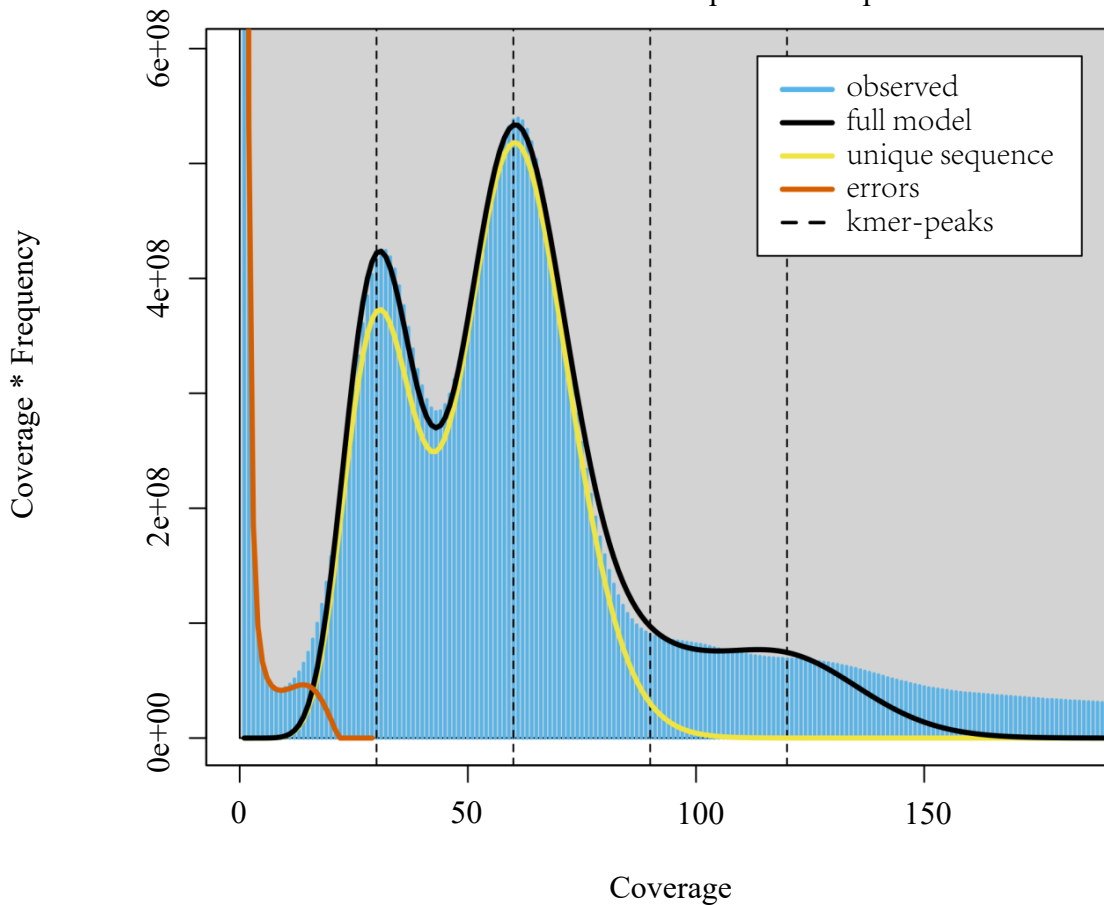

Supplement: Web_Material_uhaf156 [file web_material_uhaf156.zip › Figure S1. The 21 k-mer curve of Q. fabri genome.pdf]

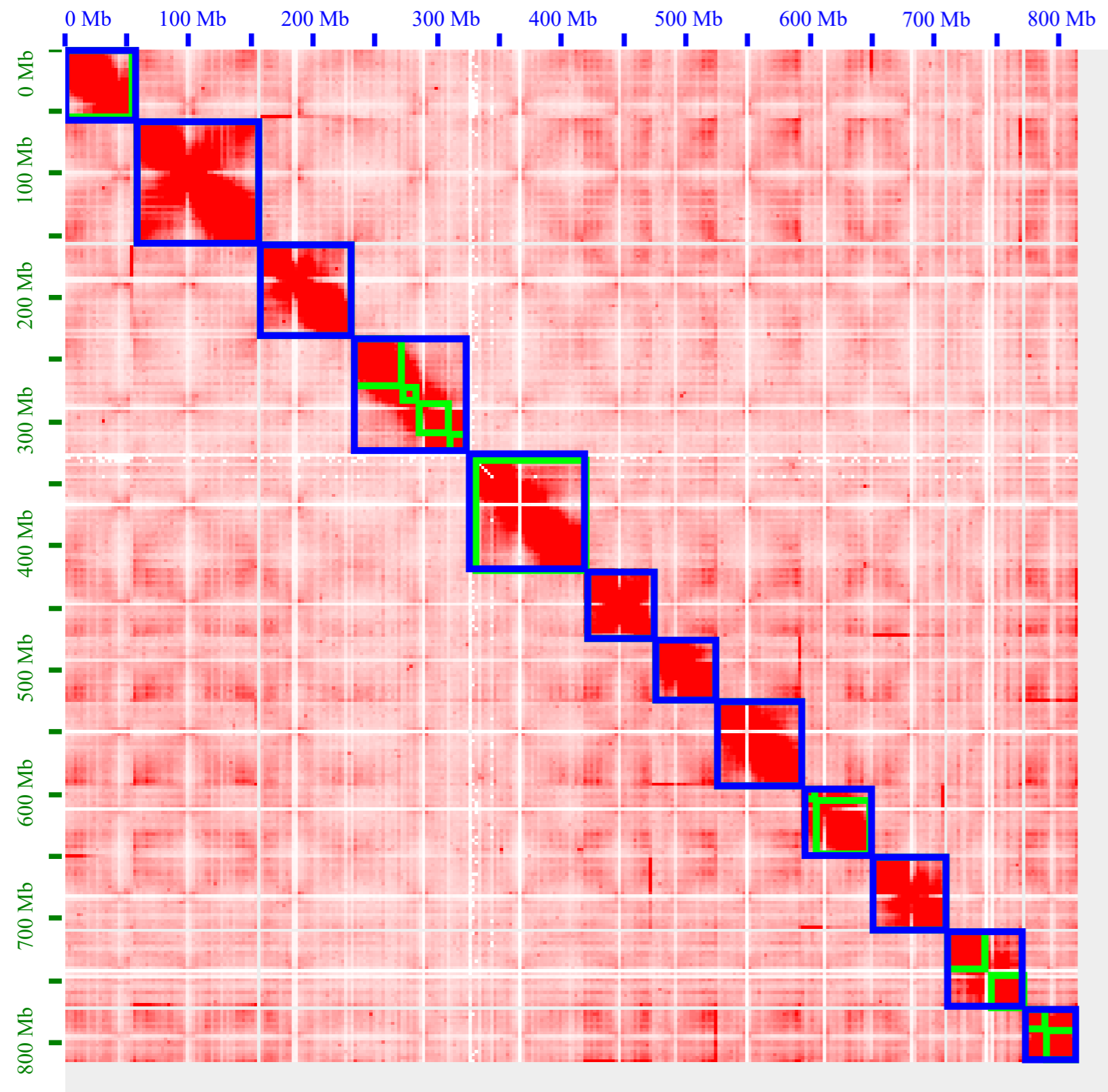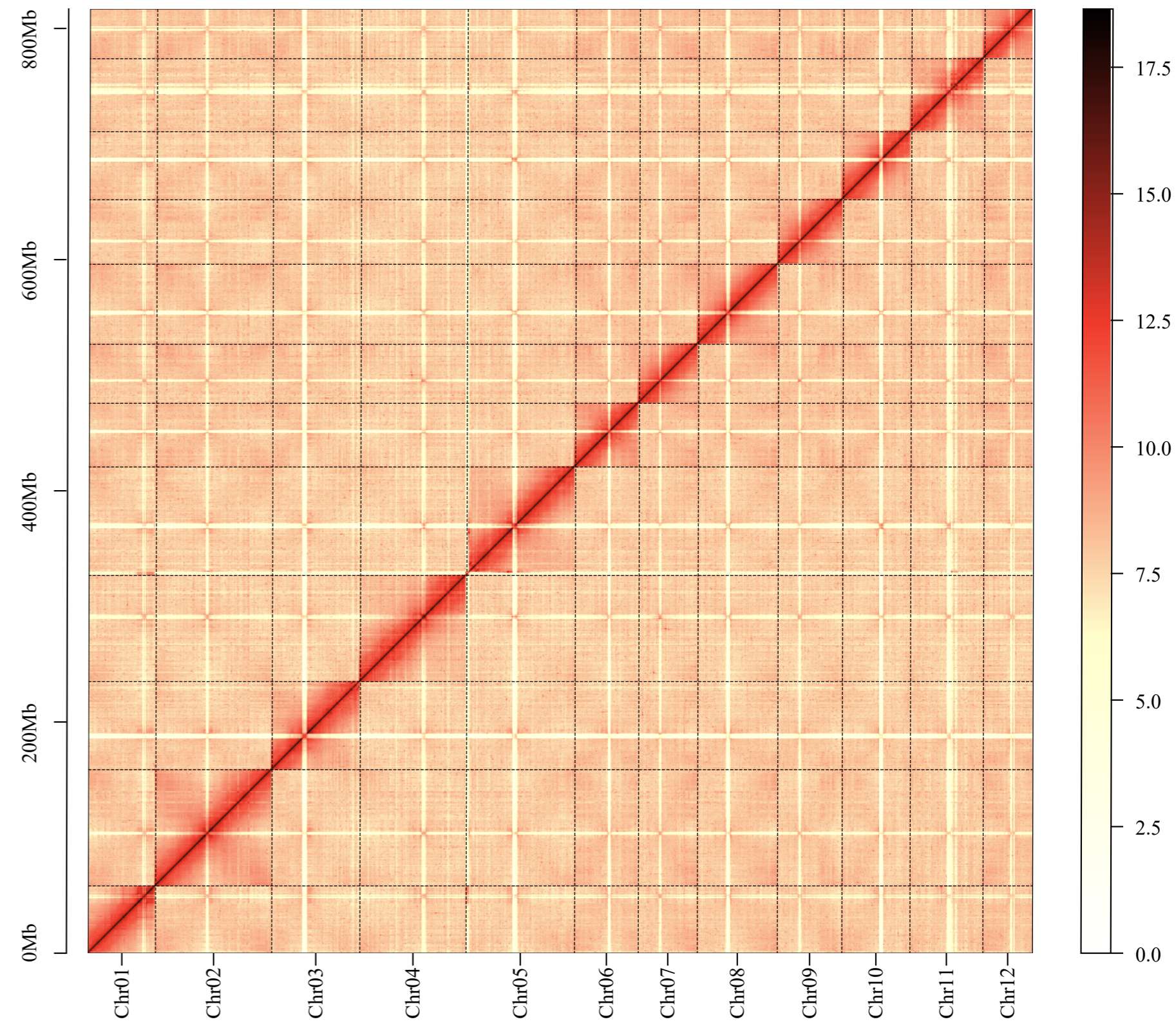

Supplement: Web_Material_uhaf156 [file web_material_uhaf156.zip › Figure S2. Corrected Hi-C clustering heatmap.pdf]

# Violin Plot of Raw Values

Raw Intensity

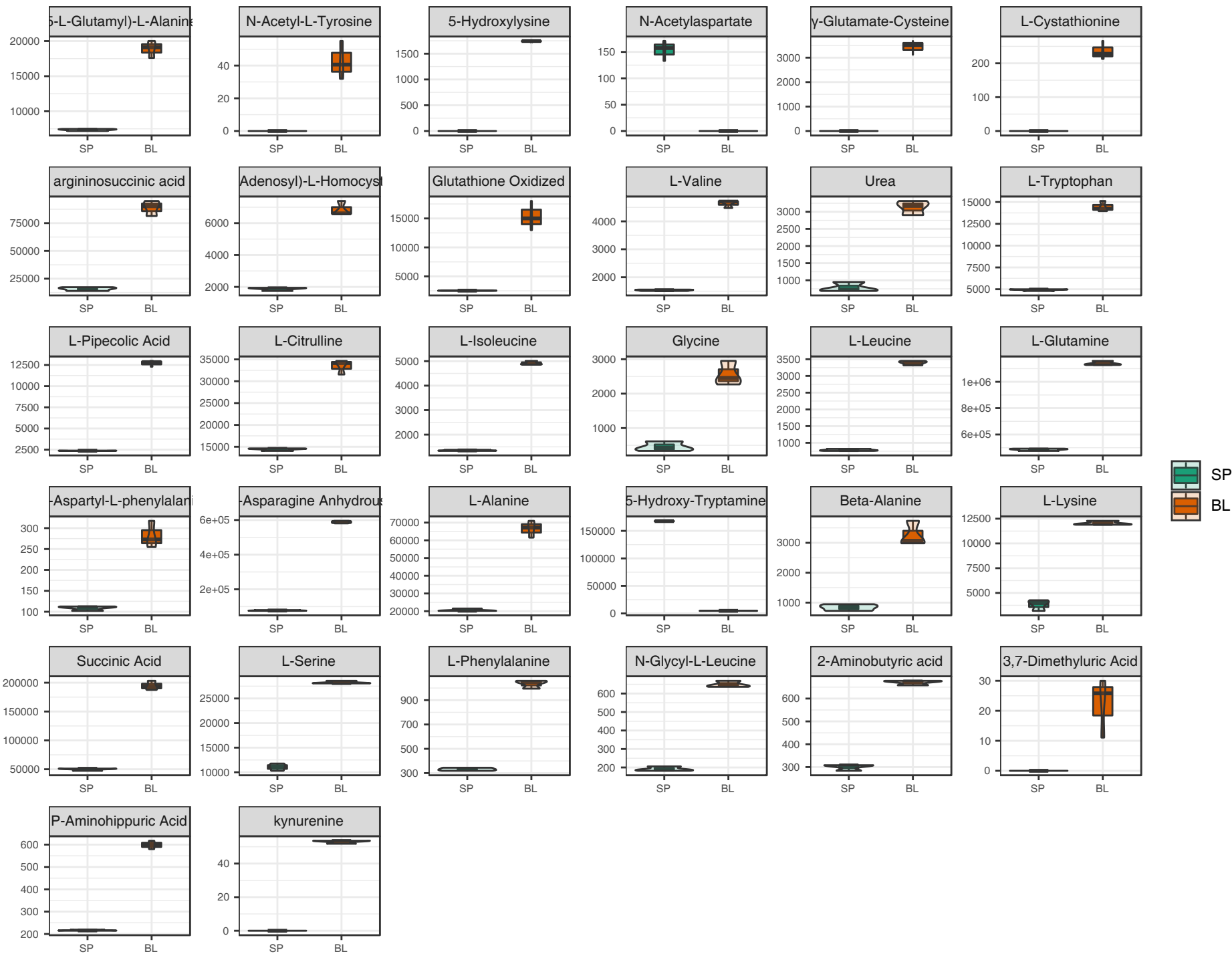

Supplement: Web_Material_uhaf156 [file web_material_uhaf156.zip › Figure S4. Amino acid comparison chart of the difference in content between Q. variabilis and Q. fabri.pdf]

Violin Plot of Raw Values

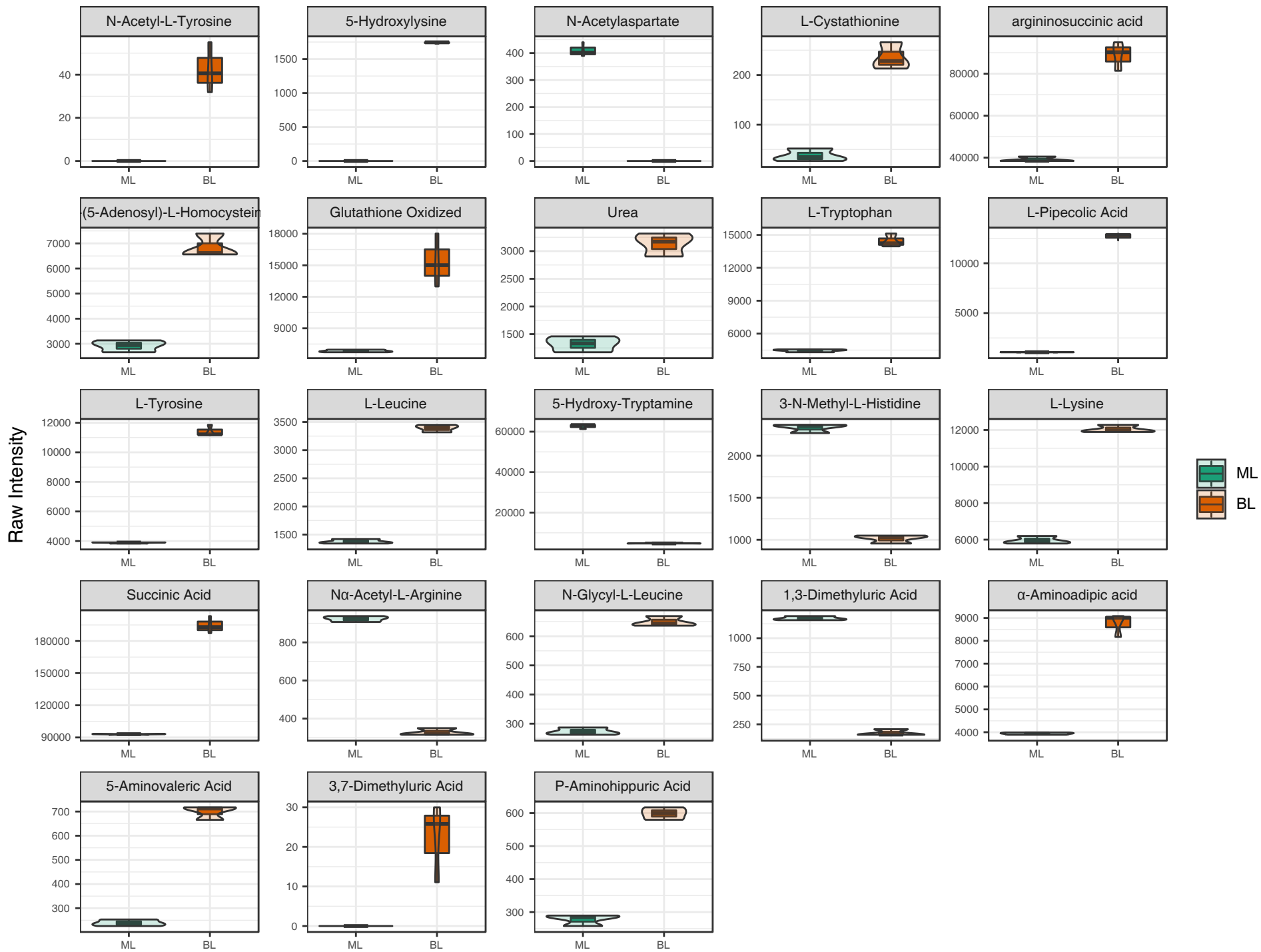

Supplement: Web_Material_uhaf156 [file web_material_uhaf156.zip › Figure S5. Amino acid comparison chart of the difference in content between Q. acutissima and Q. fabri.pdf]

# Violin Plot of Raw Values

Raw Intensity

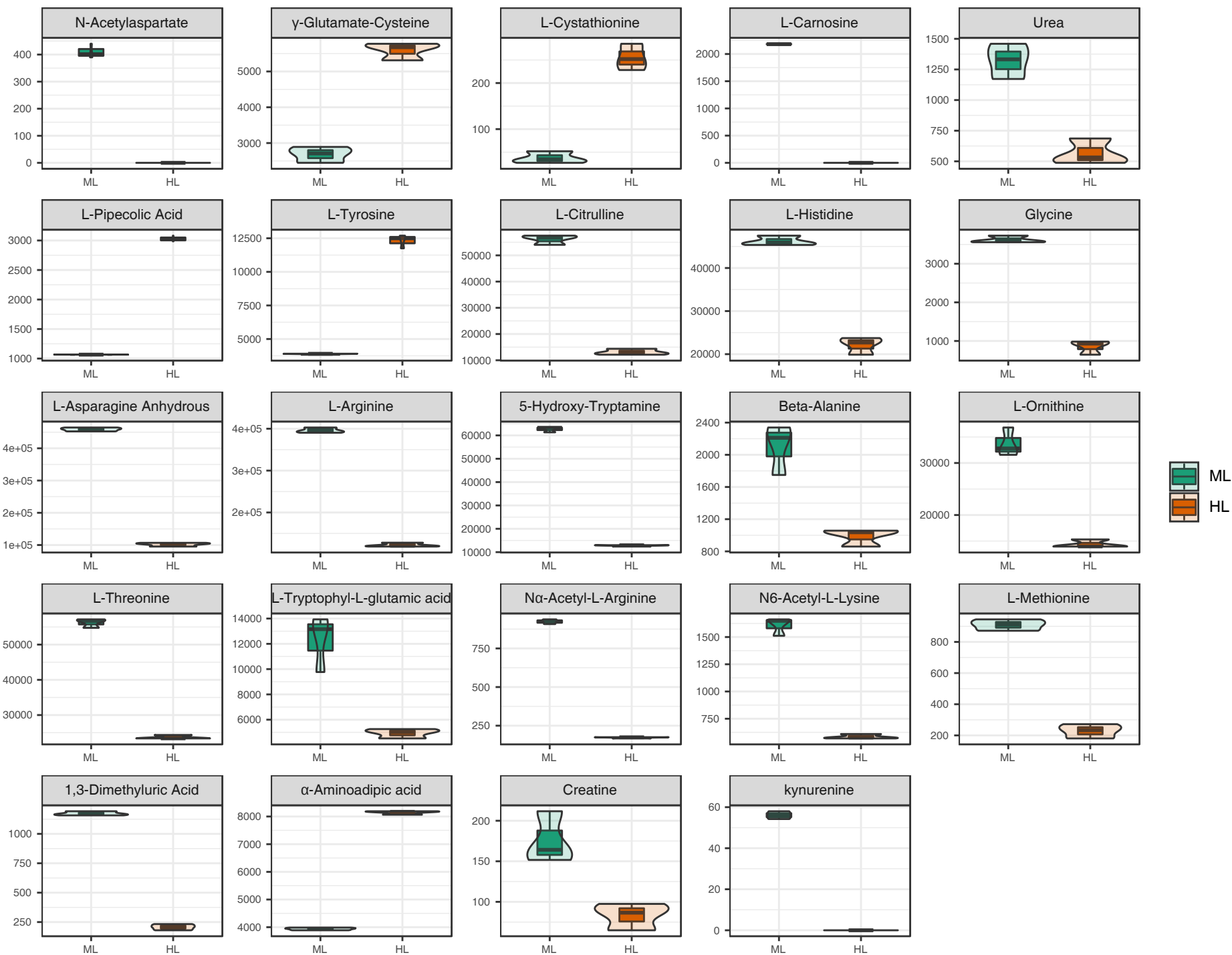

Supplement: Web_Material_uhaf156 [file web_material_uhaf156.zip › Figure S6. Amino acid comparison chart of the difference in content between Q. acutissima and Q. aliena.pdf]

# Violin Plot of Raw Values

Raw Intensity

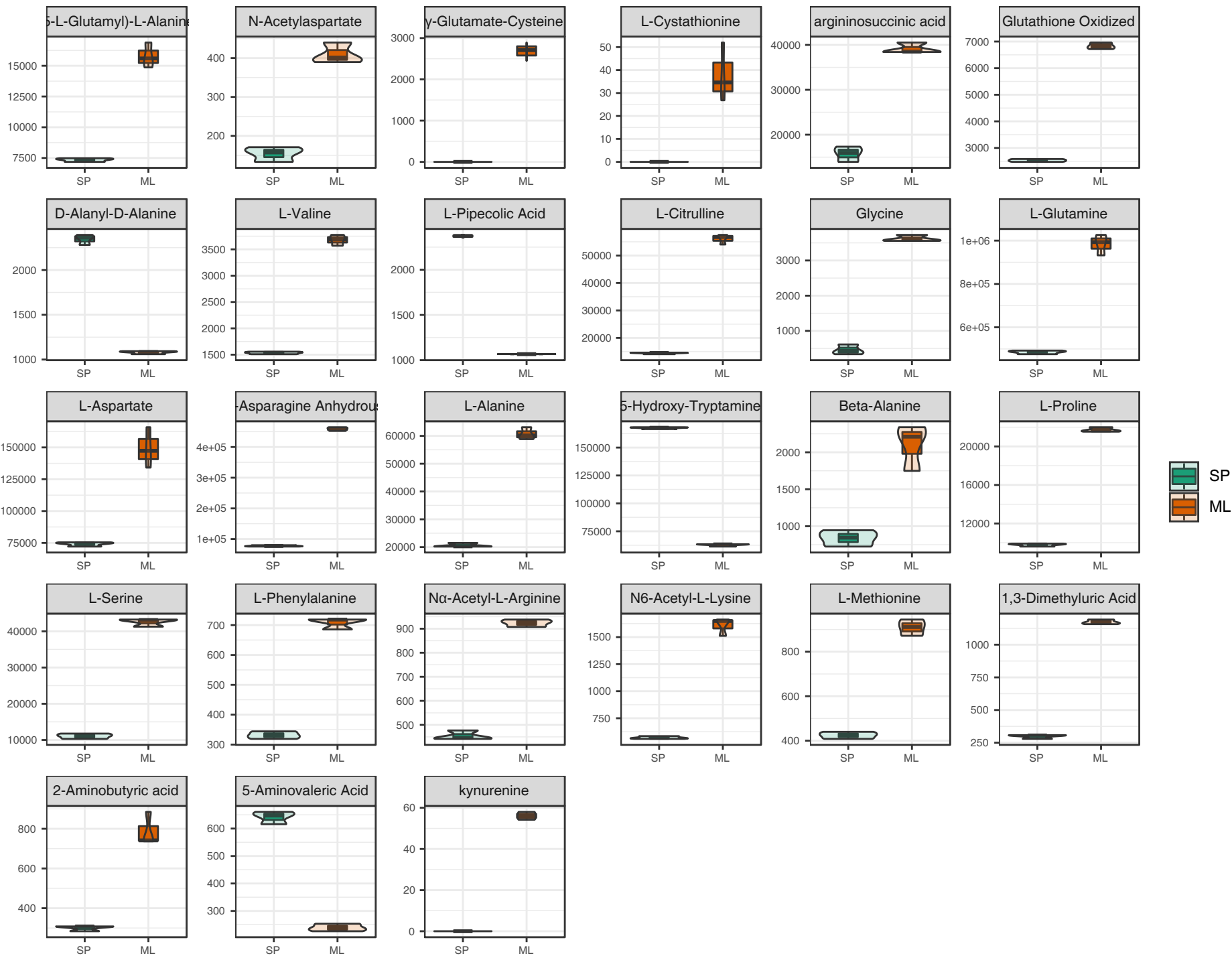

Supplement: Web_Material_uhaf156 [file web_material_uhaf156.zip › Figure S7. Amino acid comparison chart of the difference in content between Q. acutissima and Q. variabilis.pdf]
