# Supplementary material for: Uncovering the genetic basis for enhanced mushroom flavor in Quercus fabri through genome sequencing and metabolic profiling
Source: Hortic Res. 2025 Jul 9;12(9):uhaf156. doi: 10.1093/hr/uhaf156 (PMC12372586; doi:10.1093/hr/uhaf156)
Supplement: Web_Material_uhaf156 [file Figure S3. Amino acid comparison chart of the difference in content between Q. aliena and Q. fabri.pdf › Figure S3. Amino acid comparison chart of the difference in content between Q. aliena and Q. fabri.pdf]

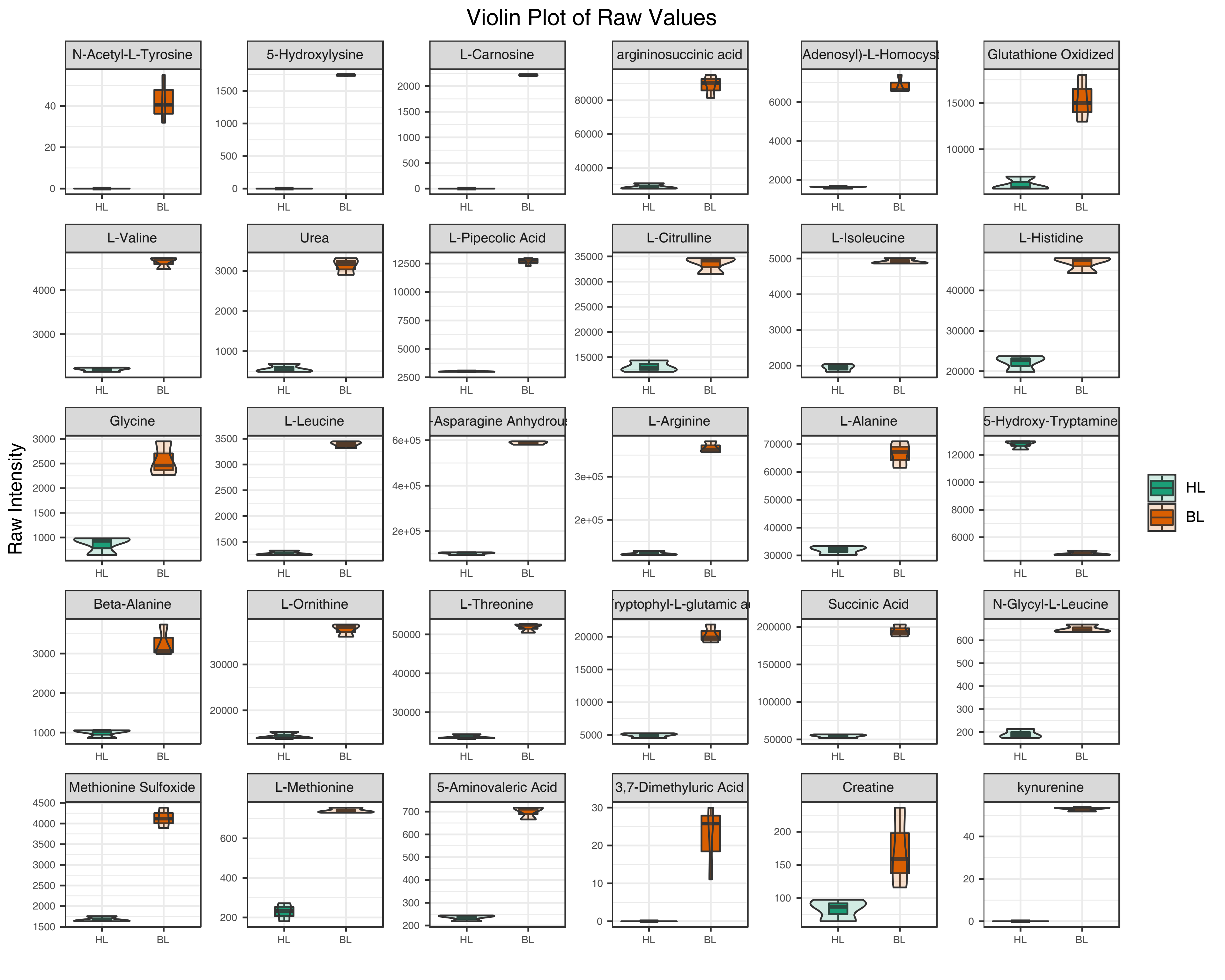
Violin Plot of Raw Values
Raw Intensity
N-Acetyl-L-Tyrosine
5-Hydroxylysine
L-Carnosine
argininosuccinic acid
Adenosyl)-L-Homocyst
Glutathione Oxidized
L-Valine
Urea
L-Pipecolic Acid
L-Citrulline
L-Isoleucine
L-Histidine
Glycine
L-Leucine
-Asparagine Anhydrou
L-Arginine
L-Alanine
5-Hydroxy-Tryptamine
Beta-Alanine
L-Ornithine
L-Threonine
ryptophyl-L-glutamic a
Succinic Acid
N-Glycyl-L-Leucine
Methionine Sulfoxide
L-Methionine
5-Aminovaleric Acid
3,7-Dimethyluric Acid
Creatine
kynurenine
HL
BL
